# Determining the incidence of heart malformations in neonates: A novel and clinically approved solution

**DOI:** 10.3389/fped.2023.1058947

**Published:** 2023-03-15

**Authors:** Arash Bordbar, Mandana Kashaki, Maryam Vafapour, Amir A. Sepehri

**Affiliations:** ^1^ Shahid Akbarabadi Clinical Research & Development Unit (ShACRDU), Iran University of Medical Sciences (IUMS), Tehran, Iran; ^2^ Department of Pediatrics, Ali-Asghar Children’s Hospital, Iran University of Medical Sciences, Tehran, Iran; ^3^ Biomedical R&D Department, CAPIS Research and Development Co., Mons, Belgium

**Keywords:** congenital heart malformations, congenital heart diseases, critical congenital heart defects, innocent murmurs, intelligent phonocardiogram, intelligent phonocardiography, Pouya Heart

## Abstract

**Background:**

Screening for critical congenital heart defects should be performed as early as possible and is essential for saving the lives of children and reducing the incidence of undetected adult congenital heart diseases. Heart malformations remain unrecognized at birth in more than 50% of neonates at maternity hospitals. Accurate screening for congenital heart malformations is possible using a certified and internationally patented digital intelligent phonocardiography machine. This study aimed to assess the actual incidence of heart defects in neonates. A pre-evaluation of the incidence of unrecognized severe and critical congenital heart defects at birth in our well-baby nursery was also performed.

**Methods:**

We conducted the Neonates Cardiac Monitoring Research Project (ethics approval number: IR-IUMS-FMD. REC.1398.098) at the Shahid Akbarabadi Maternity Hospital. This study was a retrospective analysis of congenital heart malformations observed after screening 840 neonates. Using a double-blind format, 840 neonates from the well-baby nursery were randomly chosen to undergo routine clinical examinations at birth and digital intelligent phonocardiogram examinations. A pediatric cardiologist performed echocardiography for each neonate classified as having abnormal heart sounds using an intelligent machine or during routine medical examinations. If the pediatric cardiologist requested a follow-up examination, then the neonate was considered to have a congenital heart malformation, and the cumulative incidence was calculated accordingly.

**Results:**

The incidence of heart malformations in our well-baby nursery was 5%. Furthermore, 45% of heart malformations were unrecognized in neonates at birth, including one critical congenital heart defect. The intelligent machine interpreted innocent murmurs as healthy heart sound.

**Conclusion:**

We accurately and cost-effectively screened for congenital heart malformations in all neonates in our hospital using a digital intelligent phonocardiogram. Using an intelligent machine, we successfully identified neonates with CCHD and congenital heart defects that could not be detected using standard medical examinations. The Pouya Heart machine can record and analyze sounds with a spectral power level lower than the minimum level of the human hearing threshold. Furthermore, by redesigning the study, the identification of previously unrecognized heart malformations could increase to 58%.

## 1. Introduction

Congenital heart malformations are categorized as congenital heart defects (CHD) and critical CHD (CCHD). CHD and CCHD are the leading causes of birth defect-associated illnesses and deaths of infants (1, 2) and account for 10% of all congenital anomalies (3). However, approximately 30% of mortalities attributable to congenital malformations are caused by CCHD and CHD (4). CCHD and severe CHD occur in approximately 3 out of 1,000 neonates (5, 6). Despite antenatal screening processes using fetal echocardiography, more than 50% of heart malformations remain unrecognized in neonates at birth (7, 8). Previous studies have shown that the number of adult patients with CHD is remarkably high and that we are facing a “tsunami” in terms of adult CHD cases (9–11). Consequently, timely screening of the cardiac health of neonates is vital (12, 13).

Because of the limitations of the human auditory system (e.g., masking effect) and the presence of clicks and non-pathological heart sounds, some heart abnormalities may not produce an audible murmur (14, 15); therefore, screening for heart malformations using conventional auscultation is inaccurate. Echocardiography is one of the essential modalities for the diagnosis of congenital heart disease. It must be performed by a pediatric cardiologist for the patient's management; however, the diagnostic time is relatively long. Consequently, performing a Doppler ultrasound examination for every neonate is impractical; therefore, diagnosing all neonates for detecting congenital heart malformations remains debatable (5, 16, 17).

Screening for CHD in neonates and performing echocardiography examination for positive cases is essential to saving lives and reducing the incidence of undetected pediatric and adult congenital heart diseases. Most heart sounds in neonates are inherent additional sounds that are not indications of any disease; instead, they are merely sounds rooted in the physiology of newborn hearts called innocent murmurs (15). High pulmonary pressure, high heart rates, innocent murmurs, and other factors can lead to inaccuracies when screening for congenital heart malformations in neonates using routine medical examinations, resulting in high positive and negative error rates (2, 18). Occasionally, neonates with healthy hearts are referred to the subspecialty medical departments out of an abundance of caution. The referral of children to pediatric subspecialty cardiovascular centers is a significant concern for parents (19). However, it is vital to screen for severe CHD and CCHD in neonates as early as possible (19). CCHD screening using pulse oximetry can detect approximately 50% of CCHD malformations; however, the other 50% of CCHD (such as coarctation of the aorta) are less likely to be detected by pulse oximetry in neonates (20, 21). Mandated critical CHD screening using pulse oximetry reduces early infant deaths attributable to critical CHD by 33% (22). There are other limitations when using pulse oximetry screening, such as the effect of variations in altitude among geographical locations on the oxygen level and the timing of the procedure; it is recommended that pulse oximetry screening should be performed within 24 h of birth (23).

Based on our internationally patented technology (acoustical model of the heart sound spectrum of children), we developed the first accurate intelligent digital phonocardiogram for screening CHD in children in 2008 (24). The first version required the simultaneous acquisition of electrocardiogram signals to correctly segment the heart sounds of children. However, we have developed a new algorithm for the automatic segmentation of heart sounds of children without using electrocardiogram signals (25). The sensitivity, specificity, and accuracy of an intelligent machine for the detection and discrimination of cardiac murmurs were first published in 2013 (3). The first robust and commercialized intelligent phonocardiogram for automatically screening congenital heart malformations of children (aged 28 days to 12 years) became available for purchase in 2016 (26). After the publication of the report on our internationally patented, acoustical, mathematical model of the heart sound spectrum of children, some medical universities, in conjunction with engineering schools, have developed research versions of the intelligent phonocardiogram and validated its proper functioning (3, 27–31).

The actual incidence of heart malformations (CHD and CCHD) in neonates is subject to social and economic factors (e.g., gestational diabetes in mothers creates the risk of heart malformations in their children). The Doppler ultrasound examination allows accurate screening and diagnosis of CHD in newborns; however, considering the timing of the examinations and that the echo must be done by a pediatric cardiologist, performing this examination for all neonates is impractical, especially in less privileged areas. However, a precise screening for congenital heart malformations in neonates and referring the positive cases to the subspecialist is now possible using a neonatal version of the certified and internationally patented digital intelligent phonocardiography machine known as Pouya Heart.

This study aimed to assess the actual incidence of newborn heart malformations in our well-baby nursery. In addition, our well-baby nursery also performed a pre-evaluation of the incidence of unrecognized severe and critical CHD at birth. This is the first study to evaluate the incidence of heart malformations in neonates using intelligent phonocardiography.

## 2. Materials and methods

### 2.1. Study setting

The obstetrics and gynecology departments of our hospital are accredited for the 4-year residency period and are considered the largest specialized centers in the field in Iran. Our hospital is a tertiary referral center for patients requiring gynecological and neonatal services. Furthermore, it has the country's largest and most advanced neonatal intensive care unit, with 60 active beds. Approximately 12 neonates are born daily at our hospital. Each neonate undergoes a routine medical examination, including heart auscultation, which is performed by a neonatologist within the first 24 h of birth and again before discharge. The neonatologist used a Littman Classic II stethoscope (3M, St. Paul, MN). Newborns with possible CCHD or other types of disease requiring special care are transferred to the neonatal intensive care unit, whereas healthy neonates are transferred to the well-baby nursery. Those with possible CHD are also transferred to the well-baby nursery for an echocardiography examination. Echocardiography is also performed for neonates with gestational ages <34 weeks, those with weight <1,500 g, and those whose mothers had gestational diabetes or any other illness associated with the risk of causing heart malformations in their children.

### 2.2. Study design and population

This was a retrospective analysis of 840 neonates. Neonates were randomly selected from the well-baby nursery to undergo intelligent phonocardiogram screening in a double-blinded manner. Informed consent was obtained from their legal guardians according to the study protocol. The local ethics committee of the Shahid Akbarabadi Clinical Research and Development Unit approved the study (approval number: IR.IUMS.FMD.REC.1398.098), which was conducted according to the guidelines of the World Medical Association and Declaration of Helsinki.

A pediatric cardiologist performed echocardiography to obtain a diagnosis for each neonate classified as having abnormal heart sounds by the intelligent machine. The dataset included the following: pulse rate and strength; neonatologist auscultation results; results of pulse oximetry testing of four limbs; family medical history; intelligent phonocardiography examination results; echocardiography diagnosis results.

### 2.3. Intelligent phonocardiography machine

The intelligent phonocardiogram comprises an electronic stethoscope, a medical computer cart, and a medical monitor (25). It is controlled by an operator who must have a bachelor's degree in nursing or midwifery and has undergone 60 h of training on the use of the machine. The technician records 10 s of heart sounds from two different areas of one of the four thoracic sites of the newborn using the digital stethoscope of the intelligent machine. The results appear on the screen as normal or abnormal in real-time and on a printout. The heart sounds are recorded in a room with reduced background noise; there is no time limitation when performing the intelligent machine examination. Two consecutive examinations require an average of approximately 6 min. The machine considers the heart sounds healthy if the diagnostic results at both thoracic sites are normal. In addition, the intelligent machine automatically interprets innocent murmurs as healthy sounds (3, 24, 25).

### 2.4. Neonatal version of the intelligent phonocardiography machine: Pouya Heart

The intelligent phonocardiogram, Pouya Heart, comprises passive technology for the accurate screening of CHD and CCHD in neonates. It is based on our internationally patented technology (European patent number: 2249707; United States patent number: 8,649,854; Indian patent number: 333694; Iranian patent number: 139/02/25-69899; Russian patent number: 2512794; Chinese patent number: 1045312; *Patent* Cooperation Treaty number: EP2009/051410). The operating principle of the Pouya Heart machine is based on the spectral analysis of the heart sounds of neonates on an acoustical, mathematical model of the heart sound spectrum of neonates in conjunction with advanced artificial intelligence and machine-learning techniques (24). The intelligent machine classifies heart sounds into normal or abnormal in real-time. The minimum hearing level of the human ear differs depending on the frequency. Therefore, the Pouya Heart machine can record and analyze sounds with a spectral power level lower than the minimum level of the human hearing threshold.

The Pouya Heart machine is an intelligent heart sound spectrometer for neonates. A prism is an optical spectrometer that separates photons according to their frequencies and produces red, green, and blue lights. Any lesions, weaknesses, or stiffness in the heart, organs, or blood circulation have a spectral influence on the heart sound spectrum of neonates. When using auscultation for the heart of the neonate, a patent foramen ovale (PFO) is practically inaudible; however, the incidence of a small PFO is relatively high for newborns (32, 33). A PFO influences the heart sound spectrum; therefore, the machine can be set to detect the PFO by spectrally analyzing the heart sounds. The Pouya Heart machine has received approval and certifications from the Iranian Society of Neonatology and the Ministry of Health and Medical Education.

## 3. Results

Screening using Pouya Heart was performed for 840 neonates with a median age of 30.85 h and a median weight of 3.12 kg to detect congenital heart malformations. This resulted in detecting abnormal heart sounds in 43 neonates, including one CCHD, one severe CHD, and one false-positive error. The screening and diagnosis results of the 42 neonates with congenital heart malformations identified by echocardiography are shown in Table 1.

**Table 1:** Data of 42 patients who underwent screening during the study.

Patient	Sex	Weight (kg)	Age (hr)	Apgar score at 1 min	Color of neonate	Heart bpm	Breaths per min	Pulse of limbs	Pulse oximetry	Auscultation	Family history	Pouya Heart results	Echocardiography report	Follow-up
1	M	4.15	14	9/10	Pink	137	40	NL Sym.	99	_		Abnormal	PFO/small PDA	1 week
2	M	3.65	24	9/10	Pink	143	58	NL Sym.	97	_		Abnormal	PFO/mild HC	1 week
3	M	2.15	26	9/10	Pink	157	56	NL Sym.	99	_		Abnormal	PFO/small PDA	1 week
4	F	3	12	9/10	Pink	156	44	NL Sym.	99	_		Abnormal	PFO/small PDA/mild HC	1 week
5	M	3.2	24	9/10	Pink	152	55	NL Sym.	98	_		Abnormal	Small ASD	3 months
6	M	2	96	9/10	Pink	147	43	NL Sym.	97	_		Abnormal	PDA/PFO	1 week
7	M	2.8	48	9/10	Pink	128	55	NL Sym.	99	_		Abnormal	PFO	1 week
8	M	1.55	14	9/10	Pink	154	60	NL Sym.	98	_		Abnormal	Small PDA	1 week
9	M	3	24	8/10	Pink	137	58	NL Sym.	98	_		Abnormal	mild LPA stenosis	1 week
10	M	3.4	72	9/10	Pink	123	55	NL Sym.	97	_		Abnormal	PFO/LVH	1 week
11	F	2.9	24	9/10	Pink	148	52	NL Sym.	94 to >97	_		Abnormal	Small PDA	1 week
12	M	2.8	12	8/10	Pink	144	54	NL Sym.	96	_		Abnormal	COA	NICU
13	M	2.55	24	9/10	Pink	129	46	NL Sym.	98	_		Abnormal	PFO/mild HC	1 week
14	F	3.15	4	8/10	Pink	154	43	NL Sym.	97	_		Abnormal	PFO/mild TR	1 week
15	M	1.6	168	9/10	Pink	141	49	NL Sym.	99	_		Abnormal	Small ASD	3 months
16	F	2.6	48	8/10	Pink	153	43	NL Sym.	99	_		Abnormal	Small ASD	3 months
17	M	3.45	24	8/10	Pink	141	34	NL Sym.	98	_		Abnormal	Mild LPA stenosis	1 week
18	M	3.4	18	9/10	Pink	137	56	NL Sym.	98	_		Abnormal	PFO/mild TR	1 week
19	M	2/9	24	9/10	Pink	143	42	NL Sym.	98	_		Abnormal	Small ASD, mild TR	3 months
20	M	1.85	48	8/10	Pink	145	58	NL Sym.	97	_	HTN, Diabet.	Abnormal	Small PDA	1 week
21	M	1.7	48	9/10	Pink	156	53	NL Sym.	98	_	HTN	Abnormal	Small PDA	1 week
22	M	2.9	48	9/10	Acrocyanosis	145	56	NL Sym.	97	_	Breech birth	Abnormal	Small ASD	3 months
23	M	3.3	24	8/10	Pink	148	42	NL Sym.	98	_	Diabet.	Abnormal	PFO/small PDA	1 week
24	M	3.2	24	9/10	Pink	153	46	NL Sym.	97	murmur	Diabet.	Abnormal	PFO	1 week
25	M	3	48	8/10	Pink	144	46	NL Sym.	97	murmur		Abnormal	Small PDA	1 week
26	F	3.4	48	9/10	Acrocyanosis	133	55	NL Sym.	98	_	Diabet.	Abnormal	PDA/PFO	1 week
27	M	2.75	168	9/10	Pink	161	43	NL Sym.	98	_	HTN, Diabet.	Abnormal	PFO/small PDA	1 week
28	F	2.95	18	8/10	Pink	133	50	NL Sym.	97	murmur		Abnormal	PFO/small PDA	1 week
29	M	3.6	24	9/10	Pink	148	55	NL Sym.	97	murmur		Abnormal	PFO/small PDA/mild HC	1 week
30	F	3.2	15	9/10	Pink	148	51	NL Sym.	98	_	Diabet.	Abnormal	LVH	1 week
31	M	2.55	6	9/10	Pink	156	61	bound.	97	murmur		Abnormal	PDA/LVH/HC	1 week
32	M	3.6	72	9/10	Pink	141	49	NL Sym.	98	murmur		Abnormal	LVH	1 week
33	M	3.3	14	9/10	Pink	133	52	NL Sym.	97	murmur		Abnormal	HC/IVS	1 week
34	F	3.4	24	9/10	Pink	128	55	NL Sym.	97	murmur		Abnormal	Mild LVH/mild HC	1 week
35	M	2.1	48	9/10	Pink	143	39	NL Sym.	98	_	Breech birth	Abnormal	Mild TR	1 week
36	F	4	48	9/10	Pink	130	45	NL Sym.	99	_	Diabet.	Abnormal	Mild LVH/mild HC	1 week
37	F	1/25	10	9/10	Pink	134	49	NL Sym.	96	murmur		Abnormal	Large PFO/mid mmc VSD/LR shunt	NICU
38	F	2/7	72	9/10	Pink	141	54	NL Sym.	97	_	Diabet.	Abnormal	PDA, mild L to R shunt	1 week
39	F	3/8	24	8/10	Pink	123	53	bound.	97	_	Diabet.	Abnormal	PDA, continuous shunt	1 week
40	M	3/8	24	9/10	Pink	141	58	NL Sym.	97	_		Abnormal	Small ASD	3 months
41	M	2/6	48	9/10	Pink	144	43	NL Sym.	98	_	Diabet.	Abnormal	Small VSD	2 months
42	F	3/8	120	8/10	Pink	132	43	NL Sym.	95	_	Diabet.	Abnormal	PDA + MA	5 days

Cells in pink indicate cases recognized only by the Pouya Heart examination. Cells in blue indicate cases that were recognized by the Pouya Heart and routine clinical examinations. ASD, atrial septal defect; COA, coarctation of the aorta; F, female; HC, hypertrophic cardiomyopathy; HTN, hypertension; IVC, inferior vena cava; L, left; LPA, left pulmonary artery; LVH, left ventricular hypertrophy; M, male; MA, mitral atresia; mmc, myelomeningocele; PDA, patent duct arteriosus; PFO, patent foramen ovale; R, right; TR, tricuspid valve regurgitation; VSD, ventricular septal defect.

Based on the routine medical examination results, 45% of the abnormalities observed in 42 neonates, including one CCHD, were unrecognized at birth (Table 1). The incidence of congenital heart malformations in our well-baby nursery was 5%. Routine medical examination was unable to recognize one case of CCHD at birth (Table 1). The routine medical examination results of this neonate with a CCHD included pulse oximetry of 96%, normal auscultation results, and no family history of abnormalities. Screening using Pouya Heart requires approximately 6 min and is performed by a nurse or midwife.

The sensitivity, which indicated the precision of the false-negative results, was 100%, 97.1%, and 94.8% for the intelligent phonocardiogram examination, routine medical examination, and auscultation, respectively (Table 2). The intelligent machine examinations were performed at 1–192 h after birth, and Doppler echocardiography was performed at 2–72 h after screening using Pouya Heart.

**Table 2:** Data of the 42 patients and the sensitivity, specificity, and accuracy of the pouya heat machine, clinical routine examination, and auscultation.

Total number of neonates	840
Female neonates	414
Male neonates	426
Total neonates with abnormalities detected	42
Female neonates with abnormalities detected	13
Male neonates with abnormalities detected	29
Timing of the Pouya Heart machine examination	1–192 h after birth
**Clinical routine examination:**	
Sensitivity	97.1%
Specificity	100%
Accuracy	97.7%
**Auscultation (human ear):**	
Sensitivity	94.8%
Specificity	100%
Accuracy	96%
**Pouya Heart machine:**	
Sensitivity	100%
Specificity	99.8%
Accuracy	99.8%

Sensitivity is the ability of a test to correctly detect the abnormality. It is the proportion of the true-positive results. Specificity is the ability of a test to correctly detect healthy patients. It is the proportion of true-negative results. Accuracy is the ability of a test to correctly differentiate abnormalities and healthy patients. It is the proportion of true-positive and true-negative results.

## 4. Discussion

The intelligent phonocardiogram detected heart malformations in 43 neonates. Among these, 42 neonates had heart defects confirmed by echocardiography. The pediatric cardiologist requested follow-up examinations for all 42 neonates. However, the pediatric cardiologist performed echocardiography 48 h after auscultation using Pouya Heart. Consequently, it is possible that Pouya Heart detected trivial heart defects that self-recovered after 48 h, self-recovery of trivial CHD after 48 h is possible. The complete screening process using Pouya Heart required approximately 6 min; a trained technician operated the machine. The main cost of this study was attributable to the screening of 840 neonates with Pouya Heart and echocardiography to diagnose malformations in 43 neonates. The only alternative method of obtaining such results is the performance of 840 echocardiography examinations by a pediatric cardiologist; however, this is not practical.

This study had some limitations. First, screening for CHD and CCHD in newborns using Pouya Heart is novel; consequently, some families did not allow their newborns to undergo the recommended echocardiography examinations. Second, because of the study design, we had to replace neonates who did not undergo the echocardiography examination with randomly chosen neonates from the well-baby nursery. Eleven neonates with abnormal heart sound detected by the Pouya Heart did not undergo echocardiography; consequently, they were replaced with other neonates from the well-baby nursery. Therefore, the number of neonates with congenital heart malformations who were not detected by the routine medical examination could have increased to 58% if those 11 neonates had undergone echocardiography or if other newborns from the well-baby nursery were unavailable as replacements or if we replaced those 11 neonates with those with abnormal heart sounds recognized by the Pouya Heart (total neonates examined was 855). Third, some neonates for whom an echocardiography follow-up was recommended were referred to other subspecialist centers; some might not follow those recommendations.

Consequently, it was impossible to include the follow-up results evaluation in this study, although it would be very informative to investigate the echocardiography follow-up evaluations. Forth, screening using Pouya Heart is based on an analysis of heart sounds (3, 26). Therefore, it is important to reduce the interference of background noise. Fifth, functional murmurs have a specific spectrum and are considered healthy by Pouya Heart. Any murmur is considered functional if the murmur has the same specific spectral effect as that of the functional murmurs. However, considering the size of the data in this study and the complexities of the different congenital defects, such as the branch pulmonary artery systolic murmur that can be construed as normal if it is heard in a preterm newborn in the first few days or weeks of life, but it can also be abnormal, for example, in a neonate with Alagille syndrome, Pouya Heart may lead to some false positive. Therefore, it is important to examine such complex cases in different periods interdependently with the Pouya Heart and set the spectral pattern accordingly. The adult CHD rate increased by 5% annually (9). Using both Pouya Heart and routine medical examinations can result in the timely detection of congenital heart malformations in neonates and can considerably reduce the number of adults with CHD.

In summary, we introduced a novel, practical, and cost-effective screening method for CHD and CCHD in newborns that can be performed immediately after birth. As a result, Pouya Heart detected heart malformations, including CCHD and CHD, that were unrecognized at birth by routine medical examinations at our hospital. Moreover, Pouya Heart interpreted innocent murmurs as healthy heart sounds.

Timely screening of the cardiac health of newborns is vital. Screening for CCHD should be performed as early as possible. Furthermore, screening for CHD in neonates is essential to saving lives and reducing the incidence of undetected pediatric and adult congenital heart diseases. The Pouya Heart machine can detect more congenital heart abnormalities in neonates than routine clinical examinations because of the high pressure on the right side of the heart. With many defects and anomalies, a clear murmur is sometimes not heard because of the high pressure. This critical issue emphasizes the need to use the Pouya Heart machine for screening CHD in neonates. Screening for heart malformations in newborns using Pouya Heart will significantly improve pediatric heart healthcare and eliminate unrecognized adult CHD. This intelligent machine should be tested at various hospitals in different countries to validate its accuracy across populations.

Echocardiography examination, which must be performed by a pediatric cardiologist, is essential for simultaneous screening, diagnosis, and patient's management. The Pouya Heart machine is a screening tool for congenital heart malformations. Therefore, we can conclude that the cost of conducting 43 echocardiography examinations and 840 intelligent phonocardiogram examinations to that of conducting 840 echocardiography examinations was highly cost-effective.

## Data Availability

The original contributions presented in the study are included in the article, further inquiries can be directed to the corresponding author.
