# Correction: Determining the incidence of heart malformations in neonates: a novel and clinically approved solution

**DOI:** 10.3389/fped.2025.1742255

**Published:** 2026-01-09

**Authors:** Arash Bordbar, Mandana Kashaki, Maryam Vafapour, Amir A. Sepehri

**Affiliations:** 1 Shahid Akbarabadi Clinical Research & Development Unit (ShACRDU), Iran University of Medical Sciences (IUMS), Tehran, Iran; 2 Department of Pediatrics, Ali-Asghar Children’s Hospital, Iran University of Medical Sciences, Tehran, Iran; 3 Biomedical R&D Department, CAPIS Research and Development Co., Mons, Belgium

**Keywords:** congenital heart malformations, congenital heart diseases, critical congenital heart defects, innocent murmurs, intelligent phonocardiogram, intelligent phonocardiography, Pouya heart

**Abstract**

In the abstract, the following sentence was incorrect. “This study was a retrospective analysis of congenital heart malformations observed after screening 840 neonates.” This has been corrected to read:

“This study was a prospective analysis of congenital heart malformations observed after screening 840 neonates.”

**Text correction**

A correction has been made to the section *Materials and methods*, *2.2. Study design and population,* first paragraph:

“[*This was a prospective analysis of 840 neonates. Neonates were randomly selected from the well-baby nursery to undergo intelligent phonocardiogram screening in a double-blinded manner.*]”

The original version of this article has been updated.

